# Prehospital intravenous epinephrine may boost survival of patients with traumatic cardiac arrest: a retrospective cohort study

**DOI:** 10.1186/s13049-015-0181-4

**Published:** 2015-11-19

**Authors:** Wen-Chu Chiang, Shi-Yi Chen, Patrick Chow-In Ko, Ming-Ju Hsieh, Hui-Chih Wang, Edward Pei-Chuan Huang, Chih-Wei Yang, Kah-Meng Chong, Wei-Ting Chen, Shey-Ying Chen, Matthew Huei-Ming Ma

**Affiliations:** Department of Emergency, National Taiwan University Hospital, No. 7 Zhung-Zhan S. Road, Taipei, Zhongzheng District 100 Taiwan; Department of Surgery, Kaohsiung Armed Forces General Hospital, Kaohsiung, Taiwan; Department of Medical Education, National Taiwan University Hospital, Taipei, Taiwan

**Keywords:** Traumatic cardiac arrest (TCA), Out-of-hospital cardiac arrest (OHCA), Emergency medical service (EMS), Epinephrine (Adrenaline)

## Abstract

**Background:**

Prehospital resuscitation for patients with major trauma emphasizes a load-and-go principle. For traumatic cardiac arrest (TCA) patients, the administration of vasopressors remains under debate. This study evaluated the effectiveness of epinephrine in the prehospital setting for patients with TCA.

**Methods:**

We conducted a retrospective cohort study using a prospectively collected registry for out-of-hospital cardiac arrest in Taipei. Enrollees were ≥18 years of age with TCA. Patients with signs of obvious death like decapitation or rigor mortis were excluded. Patients were grouped according to prehospital administration, or lack thereof, of epinephrine. Outcomes were sustained (≥2 h) recovery of spontaneous circulation (ROSC) and survival to discharge. A subgroup analysis was performed by stratified total prehospital time.

**Results:**

From June 1 2010 to May 31 2013, 514 cases were enrolled. Epinephrine was administered in 43 (8.4 %) cases. Among all patients, sustained ROSC and survival to discharge was 101 (19.6 %) and 20 (3.9 %), respectively. The epinephrine group versus the non-epinephrine group had higher sustained ROSC (41.9 % vs. 17.6 %, *p* < 0.01) and survival to discharge (14.0 % vs. 3.0 %, *p* < 0.01). The adjusted odds ratios (ORs) of epinephrine effect were 2.24 (95 % confidence interval (CI) 1.05-4.81) on sustained ROSC, and 2.94 (95 % CI 0.85-10.15) on survival to discharge. Subgroup analysis showed increased ORs of epinephrine effect on sustained ROSC with a longer prehospital time.

**Conclusion:**

Among adult patients with TCA in an Asian metropolitan area, administration of epinephrine in the prehospital setting was associated with increased short-term survival, especially for those with a longer prehospital time.

## Background

The survival rate of patients with traumatic cardiac arrest (TCA) is extremely low despite progress in traumatology over the past few decades [1], especially for those with no vital signs at the scene, or those who collapse during transfer to the medical facility [2–7]. Moreover, patients with prehospital TCA are always younger adults, and the loss of their lives is an enormous economic burden to society [8, 9]. Although advanced resuscitation strategies have previously been considered ineffective for TCAs, recently several studies have demonstrated improved outcome of TCAs when certain interventions are performed immediately during prehospital resuscitation, including placement of advanced airway, large volume fluid replacement, or a prehospital procedure with external bony fixation and bandage compression for bleeding control [2, 9–11].

Among the advanced resuscitative strategies for TCA, the effect of epinephrine (adrenaline) on patient outcome is still under debate. Epinephrine has a role in advanced life support because it acts as a vasoconstrictor through both alpha-adrenoreceptors and beta-adrenoreceptors, leading to better outcome, as evidenced in animal studies, by increasing coronary perfusion and cerebral blood flow. Although a previous study using an animal model with uncontrolled hemorrhagic shock linked the use of epinephrine to worse outcomes compared to high-volume fluid resuscitation [12], a recent report supported the use of vasopressors in “buying time” for definitive treatment for uncontrolled hemorrhagic shock in rats [13]. Regarding human subjects, prehospital administration of epinephrine during cardiopulmonary resuscitation (CPR) in non-traumatic out-of-hospital cardiac arrest (OHCA) has been shown to induce improved return of spontaneous circulation (ROSC) ratio and short-term survival, but point towards either no benefit or even harm of this drug for long-term survival or functional recovery [9, 13–19]. However, a recent report in patients with non-shockable cardiac arrest in hospital, demonstrated that earlier administration of epinephrine was associated with a higher probability of ROSC, survival in hospital, and neurologically intact survival [20].

Although results from animal studies showed the potential benefit of vasopressors in hemorrhagic shock [13], and a human study found that the deficiency of vasopressin and epinephrine may contribute to intractable shock following trauma [10], there is little data focusing on the clinical outcome of patients with out-of-hospital TCA who received administration of epinephrine. To elucidate this issue, we conducted a study to evaluate the effectiveness of administration of intravenous epinephrine for patients with TCA in the prehospital setting.

## Material and methods

### Study design and setting

We conducted a retrospective cohort study using a prospectively collected Utstein-based registry system for patients with OHCA from a Taipei emergency medical service (EMS) to study the effectiveness of intravenous epinephrine for TCA and its influence among subgroups with different total prehospital times. The Utstein-based registry of Taipei EMS, which was initially developed for OHCA process quality assurance [21], consisted of: dispatch records from the Taipei City Fire Department, modes and timing of prehospital care, patient demographics (age, sex), arrest characteristics (witness status, bystander cardiopulmonary resuscitation, initial rhythm on cardiac monitor), records of whether an automated external defibrillator was used, patient records from the EMS-receiving hospitals, and patient outcomes (prehospital ROSC, sustained ROSC (≥2 h), survival to emergency department (ED)/intensive care unit (ICU) admission, survival to hospital discharge, and neurologic status on discharge) [22].

Taipei City is a metropolitan area with a registered population of 2.65 million in 272 km^2^ and up to 3.0 million including inflow daytime workers. The majority of the population is Taiwanese or Chinese. The metropolitan area is covered by a fire department–based basic life support plus defibrillator (BLS-D) system with early defibrillation capability. The service is provided by 45 ambulance teams staffed by 1020 emergency medical technicians (EMTs) who each completed at least 264 hours of training. From 2008 to 2010, the prehospital advanced life support (ALS) service covered three of the 12 administrative districts in Taipei; the coverage was extended to four of the 12 districts after 2010. The ALS service is staffed by 120 EMT paramedics who each completed, in accordance with the requirements of the Taiwan Department of Health, 1280 hours of training. The ALS providers are able and authorized to perform tracheal intubation as well as intravenous injections of medications (epinephrine, atropine, and amiodarone) for cardiac arrest. All incoming calls for EMS are processed by a central dispatch center staffed by dispatchers with 40 hours each of training in priority dispatch. For cases that originate from the catchment areas where ALS services are available, ALS services are activated in addition to BLS-D when predetermined ALS dispatch criteria are met.

### Study population

Adult patients (≥18 years) with TCA that activated EMS between June 1 2010 and May 31 2013 were included in the study. TCA was defined as a cardiac arrest that was a consequence of a prior traumatic event. Patients would not have been transported to hospital and thus were excluded from the final analysis, if they had obvious signs of death like decapitation or rigor mortis.

### Ethical considerations

The study protocol was approved by the Institutional Review Board of National Taiwan University Hospital.

### Definition of exposure and outcome

Exposure was defined as the administration of intravenous epinephrine in the prehospital setting, whether that be at the scene of the trauma or during ambulance transport. Although the administration of intravenous epinephrine is required for all non-traumatic OHCAs in line with EMTs protocol, there are no strict rules in Taipei city for the administration of epinephrine in traumatic OHCAs under the consideration of the load-and-go principle. Therefore, the decision to give epinephrine or not in patients with TCA is primarily dependent on the clinical judgment of the EMTs.

Outcomes of this study were defined as sustained (≥2 h) ROSC and survival to hospital discharge. We also explored the relationship between total prehospital time and the effectiveness of epinephrine on outcomes.

### Statistical analyses

Data were entered in Excel (Microsoft, Redmond, WA, USA) and were subsequently processed and analyzed by SAS software version 9.3 (SAS Institute, Cary, NC, USA). Descriptive statistics of the population were given as counts, percentages, or median (Q1-Q3). We used non-parametric Mann–Whitney rank sum test to compare the differences of continuous variables and the chi-square test or Fisher exact test as appropriate to assess the associations between categorical variables and the outcomes. All variables showing a possible association with outcomes (*p* < 0.05) were entered into the multivariate logistic regression analysis. Collinearity of covariates was assessed by the correlation coefficient. The model fitting was assessed by the Hosmer-Lemeshow goodness-of-fit test. Odds ratios (ORs) and 95 % confidence intervals (CIs) were calculated and two-tailed *p*-values of <0.05 were considered significant.

## Results

**Fig. 1:**
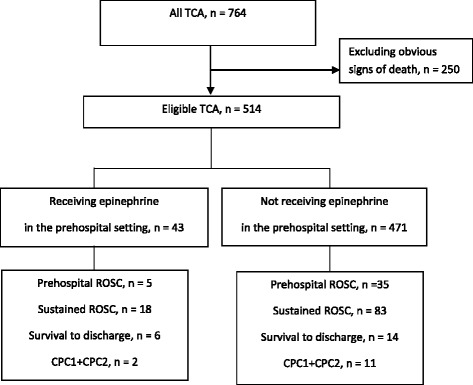
Patient flow by administration of epinephrine, TCA: traumatic cardiac arrest; ROSC: return of spontaneous circulation; CPC: cerebral performance category

From June 1 2010 to May 31 2013, there were 764 patients with TCA managed by the Taipei EMS. After excluding cases according to pre-specified criteria (*n* = 250), 514 adult cases were enrolled in the final analysis, 43 of whom received administration of intravenous epinephrine, and 471 of whom did not. Figure 1 provides an overview of TCAs evaluated during the study period.

Comparisons of demographic data and outcomes of eligible patients stratified by administration, or lack thereof, of epinephrine are shown in Table 1. Although the study was a retrospective design, the Utstein registry database was prospectively collected by EMS personnel, with regularly monitored data quality. Thus, among all variables and outcome included in the analyses, the missing rates were 0 % (most of them) to 2.6 % (variable of “total prehospital time”). There were no significant differences between the epinephrine group and non-epinephrine group regarding sex, age, administration of bystander CPR, EMS response time, EMS transport time, and destination hospital. However, the EMS scene time was longer, and the percentages of unwitnessed status, presenting shockable rhythm, mechanism by non-blunt trauma, and application of advanced airway (i.e. laryngeal mask airway or tracheal intubation) were greater in the epinephrine group than in the non-epinephrine group in univariate analysis.

**Table 1:** Comparisons of demographic data and outcomes of enrolled patients with traumatic cardiac arrest between epinephrine group and non-epinephrine group

Characteristics	All TCA	Epinephrine group	Non-epinephrine group	*p*-value
Number of cases	514	43	471	
Patient characteristics: (n (%); age: median (Q1–Q3))				
Gender-male	348 (67.7 %)	27 (62.8 %)	321 (68.2 %)	0.50
Age, median (Q1–Q3)	48.0 (30.0–64.0)	46.0 (35.0–75.0)	48.0 (30.0–63.0)	0.36
Witnessed	194 (37.7 %)	8 (18.6 %)	186 (39.5 %)	<0.01
Bystander CPR	110 (21.4 %)	13 (30.2 %)	97 (20.6 %)	0.17
Shockable rhythm	24 (4.7 %)	5 (11.6 %)	19 (4.0 %)	0.04
Blunt injury	388 (75.5 %)	11 (25.6 %)	377 (80.0 %)	<0.01
EMS variable: (time: median (Q1–Q3); n (%))				
Response time	5.0 (4.0–6.0)	5.0 (4.0–7.0)	5.0 (4.0–6.0)	0.07
Scene time	11.0 (8.0–14.0)	14.0 (12.0–17.0)	11.0 (8.0–14.0)	<0.01
Transport time	3.0 (2.0–5.0)	3.0 (2.0–5.0)	3.0 (2.0–5.0)	0.23
Total prehospital time	20.0 (16.0–25.0)	23.0 (20.0–29.0)	20.0 (16.0–25.0)	<0.01
Advance airway	124 (24.1 %)	32 (74.4 %)	92 (19.5 %)	<0.01
Destination at trauma center	349 (67.9 %)	26 (60.5 %)	323 (68.6 %)	0.31
Patient outcomes: n (%)				
Prehospital ROSC	40 (7.8 %)	5 (11.6 %)	35 (7.4 %)	0.37
Sustained ROSC	101 (19.6 %)	18 (41.9 %)	83 (17.6 %)	<0.01
Survival to discharge	20 (3.9 %)	6 (14.0 %)	14 (3.0 %)	<0.01
CPC–1 & CPC–2	12 (2.3 %)	2 (4.7 %)	11 (2.3 %)	0.30

*TCA* traumatic cardiac arrest, *EMS* emergency medical service, *CPR* cardiopulmonary resuscitation, *ROSC* return of spontaneous circulation, *CPC* cerebral performance category

Longer scene times in the epinephrine group were likely due to the time consumed for establishment of intravenous route or tracheal intubation by paramedics. More advanced airway support in the epinephrine group was because in our EMS only paramedics were authorized to perform tracheal intubation as well as intravenous injections of epinephrine. The reason for higher ratios of presenting shockable rhythm and mechanism by non-blunt trauma in the epinephrine group was uncertain. A maybe explanation was that EMT selectively administrated epinephrine among patients with more likelihood of survival according to their subjective feelings. However, correlation coefficients between receiving epinephrine and shockable rhythm, or epinephrine and non-blunt mechanism were 0.049 and 0.362, respectively. Low correlation coefficients indicated the administration of epinephrine was not severely biased by self-fulfilling prophecy of EMTs toward the patients with traumatic cardiac arrest.

**Fig. 2:**
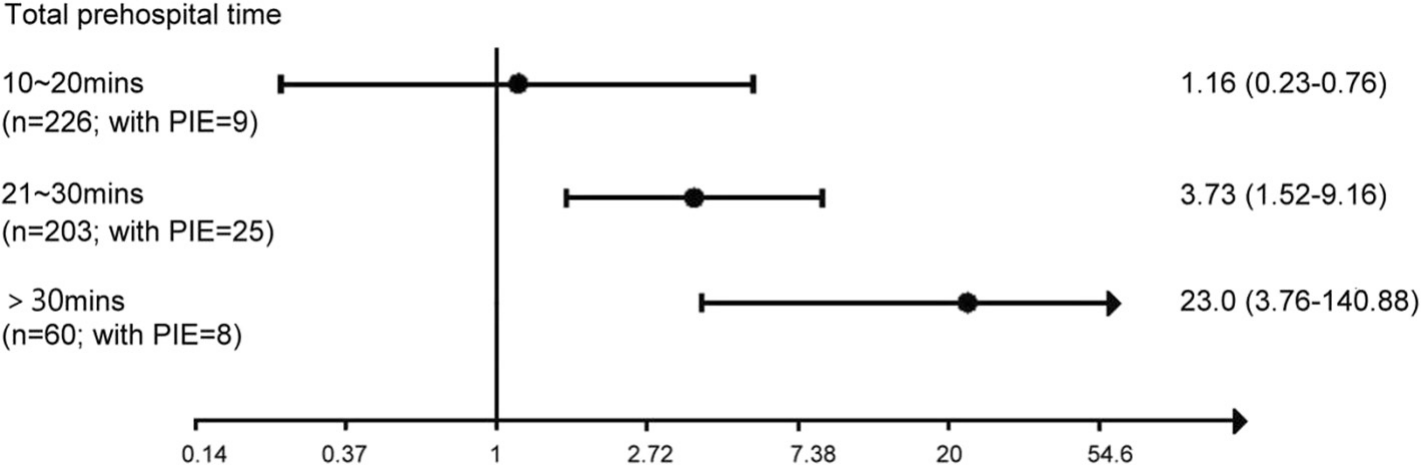
Odds ratios of epinephrine effect on sustained ROSC stratified by total prehospital time. PIE: prehospital intravenous epinephrine; ROSC: return of spontaneous circulation

The outcome of sustained ROSC (≥2 h) was significantly better in the epinephrine group versus the non-epinephrine group (41.9 % vs. 17.6 %, *p* < 0.01). The percentage of survival to discharge was also higher in the epinephrine group versus the non-epinephrine group (14.0 % vs. 3.0 %, *p* < 0.01). Favorable neurological status at one month defined by cerebral performance category (CPC) level 1 and level 2 did not differ between the two groups. Associations between variables and outcome (sustained ROSC) were assessed by unadjusted ORs of univariate analyses and adjusted ORs of unconditional logistic regression analysis, as shown in Table 2. Prehospital administration of intravenous epinephrine for patients with TCA resulted in significant improvement in the achievement of sustained ROSC (OR 2.24; 95 % CI: 1.05–4.81) after adjustment in the final model. The adjusted ORs of epinephrine effect was 2.94 (95 % CI 0.85–10.15) on survival to discharge, as shown in final model in Table 3. Hosmer-Lemeshow goodness-of-fit test shows a satisfactory model fitting (*p* = 0.07 for model of sustained ROSC and *p* = 0.42 for model of survival to discharge). For those who received prehospital administration of epinephrine, stratification analysis showed that the longer the total prehospital time, the more significant the positive effect of epinephrine on sustained ROSC, as illustrated in Fig. 2.

**Table 2:** Unadjusted odds ratios and adjusted odds ratios of administration of intravenous epinephrine on primary outcome (sustained ROSC for longer than 2 hours) among patients with traumatic cardiac arrest

Characteristics	With sustained ROSC, *n* = 101	Without sustained ROSC, *n* = 413	*p*-value	Unadjusted ORs and 95 % CI	Adjusted ORs and 95 % CI
Gender-male, n (%)	67 (66.3 %)	281 (68.0 %)	0.81	0.93 (0.58–1.47)	
Age (median (Q1–Q3))	51.0 (35.0–69.5)	46.0 (30.0–62.0)	0.04	1.01 (1.00–1.02)	1.01 (1.00–1.02)
EMS Time: (median (Q1–Q3))					
Response	4.0 (3.0–6.0)	5.0 (4.0–6.0)	0.09	0.95 (0.87–1.03)	
Scene	12.0 (8.0–15.0)	11.0 (8.0–14.0)	0.58	0.99 (0.96–1.02)	
Transport	3.0 (2.0–4.0)	3.0 (2.0–5.0)	<0.01	0.88 (0.80–0.97)	0.87 (0.79–0.97)
Total prehospital time	19.0 (15.0–26.0)	20.0 (16.0–25.0)	0.21	0.98 (0.95–1.01)	
Witness, n (%)	45 (44.6 %)	149 (36.1 %)	0.14	1.42 (0.92–2.21)	
Bystander CPR, n (%)	29 (28.7 %)	81 (19.6 %)	0.06	1.65 (1.01–2.71)	
Shockable rhythm, n (%)	13 (12.9 %)	11 (2.7 %)	<0.01	5.40 (2.34–12.45)	5.05 (2.09–12.21)
Blunt injury, n (%)	64 (63.4 %)	324 (78.5 %)	<0.01	0.48 (0.30–0.76)	0.66 (0.38–1.13)
Advance airway, n (%)	32 (31.7 %)	92 (22.3 %)	0.05	1.62 (1.00–2.61)	
Destination at trauma center, n (%)	68 (67.3 %)	281 (68.0 %)	0.91	0.97 (0.61–1.54)	
Epinephrine, n (%)	18 (17.8 %)	25 (6.1 %)	<0.01	3.37 (1.76–6.45)	2.24 (1.05–4.81)

*TCA* traumatic cardiac arrest, *ROSC* return of spontaneous circulation

**Table 3:** Unadjusted odds ratios and adjusted odds ratios of administration of intravenous epinephrine on secondary outcome (survival to discharge) among patients with traumatic cardiac arrest

Characteristics	Survival to discharge, *n* = 20	Mortality at discharge, *n* = 494	*p*-value	Unadjusted ORs and 95 % CI	Adjusted ORs and 95 % CI
Gender-male, n (%)	16 (80.0 %)	332 (67.2 %)	0.33	1.95 (0.64–5.93)	
Age (median (Q1–Q3))	50.0 (35.0–72.0)	48.0 (30.0–64.0)	0.56	1.01 (0.99–1.03)	
EMS Time: (median (Q1–Q3))					
Response	5.0 (3.0–6.0)	5.0 (4.0–6.0)	0.55	0.90 (0.74–1.11)	
Scene	11.0 (8.0–14.5)	11.0 (8.0–14.0)	0.93	0.99 (0.93–1.06)	
Transport	2.0 (2.0–5.5)	3.0 (2.0–5.0)	0.32	0.93 (0.77–1.11)	
Total prehospital time	18.0 (14.5–28.0)	20.0 (16.0–25.0)	0.54	0.98 (0.93–1.04)	
Witness, n (%)	9 (45.0 %)	185 (37.5 %)	0.49	1.37 (0.56–3.36)	
Bystander CPR, n (%)	6 (30.0 %)	104 (21.1 %)	0.40	1.61 (0.60–4.28)	
Shockable rhythm, n (%)	9 (45.0 %)	15 (3.0 %)	<0.01	26.1 (9.42–72.45)	23.29 (8.08–67.15)
Blunt injury, n (%)	10 (50.0 %)	378 (76.5 %)	0.01	0.31 (0.12–0.76)	0.45 (0.16–1.31)
Advance airway, n (%)	7 (35.0 %)	117 (23.7 %)	0.28	1.74 (0.68–4.45)	
Destination at trauma center, n (%)	16 (80.0 %)	333 (67.4 %)	0.33	1.93 (0.64–5.88)	
Epinephrine, n (%)	6 (30.0 %)	37 (7.5 %)	<0.01	5.29 (1.92–14.58)	2.94 (0.85–10.15)

*TCA* traumatic cardiac arrest, *ROSC* return of spontaneous circulation

## Discussion

In our study, we found a strong positive association between receiving intravenous epinephrine in the prehospital setting and improved survival in patients with TCA. This positive association remained consistent after multivariate analyses, and was more significant in a subgroup with longer total prehospital time. To the best of our knowledge, this association has not been widely investigated before. Our study suggests that use of vasopressors for TCAs in the prehospital setting is beneficial.

In the past, emergency physicians and trauma surgeons have not favored the use of vasopressors as the first line treatment in patients with major trauma, because of the risk of increasing the amount of bleeding and of rebounded shock after bleeding has been controlled [23]. Bleeding control and volume replacement is one of the major concepts of resuscitation for patients with major trauma in hospital. However, the concept has not been validated in patients with TCA in the prehospital setting. Patients with TCA at roadside have a limited numbers of EMTs to perform resuscitation, and an extremely short therapeutic window to regain the vital signs (i.e. the very first step to survive). The hemostasis and vigorous fluid challenge at the scene is a time-consuming job requiring considerable manpower. Administration of epinephrine might boost peripheral blood reserve to vital organs, and thus increase the chance of survival of TCA, as evidenced in our study. Lacking of nationwide registry for patients with trauma, we cannot exactly calculate the incidence of TCA, but the survival rates of TCA in our study (i.e. 19.6 % of sustained ROSC and 3.9 % of survival to discharge) were not lower comparing to previous literature [2].

A study by Grmec et al., although not conducted in prehospital patients with TCA as in our study, suggested that a treatment protocol including vasopressor and hydroxyethyl starch solution was associated with increased ROSC in blunt trauma patients with pulseless electrical activity [24]. The effect of vasopressor therapy for TCA is still under debate. There are some viewpoints supporting the positive association of prehospital intravenous epinephrine and higher chance of survival observed in our study. The most common cause of TCA in the prehospital setting is due to loss of effective cardiac output because of a loss of preload caused by hypovolemic shock or obstructive shock [25, 26]. There are two phases of physiopathological response to acute hemorrhage [27]. The early phase is characterized by sympathetic system activation resulting in vasoconstriction to normalize blood pressure. In the late phase, after a certain amount of preload decrease, sympathetic tone becomes inadequate, leading to a drop in vascular resistance and bradycardia, which might rapidly proceed to cardiac arrest. Theoretically, the use of vasopressors in the late stage would provide benefit to insufficient systemic vasoconstrictions, and may be useful for restoring hemodynamic parameters and reducing the need of large amount of fluid infusion, which lead to side effects such as tissue edema. This theory provides plausibility to the observation in our study, which suggested that the effect of prehospital intravenous epinephrine is even more obvious in cases with a longer prehospital time.

Despite the interesting findings depicted by this study, some limitations deserve careful considerations, and the results may not be applicable to all countries and EMSs. First of all, our study has all the inherent problems associated with retrospective design. Some sort of selection bias or the effect from other potential confounders, such as patient preceding comorbidity, in-hospital trauma care, or post-resuscitation care, could not be adjusted for, although the percentage of TCA among the epinephrine group versus the non-epinephrine group treated in the trauma center had no statistical difference. Some ongoing studies, such as one European trial (VITRIS-vasopressin for therapy of persistent traumatic hemorrhagic shock; National Clinical Trial number 00379522), may provide more robust evidence to this issue.

Second, we do not have records of the exact amounts of intravenous fluid provided during resuscitation of patients with TCA in the prehospital setting. However, because data used in this study were collected in a metropolitan area with many tertiary hospitals, the average transport time was relatively short (4.1 ± 3.6 minutes) and therefore, the actual amount of challenging fluid was always estimated to be less than a pack of saline (i.e. 500 ml). Third, there is some chance that cases categorized as TCA at the scene might have resulted from a pre-existing medical cardiac arrest. Some experts suggested the presenting rhythms with ventricular arrhythmias are more likely in medical cardiac arrest then in TCA [8]. However, in our area, EMS protocol regulates paramedics to give intravenous epinephrine to all cases of OHCA, whether they are medical or traumatic cases, so the misclassification of a non-TCA as a TCA should be a non-directional error in this study. We did have more ventricular arrhythmias in the epinephrine group. However, the effect of epinephrine on patient survival remained significant after adjustment for shockable rhythms. Finally, the lack of long-term outcomes makes the evidence found in this study not that robust. Long-term outcomes are of paramount importance after injury. However, in our current database from June 1 2010 and May 31 2013, we did not have the information. We are now setting up a newly developed trauma registry in Taipei City, in which we will collect functional status at the 6th month and 12th month after injury in the future.

## Conclusion

In summary, among patients with TCA in an Asian metropolitan area, administration of intravenous epinephrine in the prehospital setting was associated with higher sustained ROSC and survival to discharge, especially for those with longer prehospital time.

## Abbreviations

ALS: Advanced life support
BLS-D: Based basic life support plus defibrillator
CPC: Cerebral performance category
CPR: Cardiopulmonary resuscitation
EMS: Emergency medical service
OHCA: Out-of-hospital cardiac arrest
PIE: Prehospital intravenous epinephrine
ROSC: Return of spontaneous circulation
TCA: Traumatic cardiac arrest
